# Mechanical stretching of the pulmonary vein mediates pulmonary hypertension due to left heart disease by regulating SAC/MAPK pathway and the expression of IL-6 and TNF-α

**DOI:** 10.1186/s13019-021-01471-5

**Published:** 2021-05-10

**Authors:** Wenhui Huang, Hongjin Liu, Yichao Pan, Hongwei Yang, Jing Lin, Hui Zhang

**Affiliations:** 1 Department of Cardiovascular Surgery, Union Hospital, Fujian Medical University, Fuzhou, 350001 Fujian Province People’s Republic of China; 2 Anesthesiology Research Institute, the First Affiliated Hospital, Fujian Medical University, Fuzhou, 350004 Fujian Province People’s Republic of China; 3 Department of Intensive Care Unit, Union Hospital, Fujian Medical University, Fuzhou, 350004 Fujian Province People’s Republic of China

**Keywords:** Pulmonary hypertension due to left heart disease, Pulmonary vein, MAPKs, IL-6, TNF-α

## Abstract

**Background:**

This study aimed to explore whether the mechanical stretching-induced expression of interleukin-6 (IL-6) and tumor necrosis factor-α (TNF-α) in pulmonary veins occurred through the stretch-activated channel (SAC)/ mitogen-activated protein kinases (MAPKs) pathway.

**Methods:**

Sixty male Sprague-Dawley rats were divided into three sham groups and seven model groups. A metal clip was placed on the ascending aorta in the model group to establish PH-LHD rat model. The sham group received a similar operation without ascending aorta clamped. On day 25, pulmonary vein was given mechanical stretching with 0 g, 2.0 g tension in two model groups and two sham groups. Another four model groups were given 2.0 g tension after MAPKs pathway inhibitors soaked. The last sham group and model group rats’ pulmonary veins, pulmonary artery and lung tissues were obtained on day 35. Pulmonary vein, pulmonary artery and lung tissue were evaluated by echocardiography, HE staining, immunohistochemistry and western blotting respectively.

**Results:**

On day 25, left heart weight, right ventricular pressure (35.339 cmH_2_O) and left atrial pressure (13.657 cmH_2_O) were increased in model group than those in sham group. Echocardiography showed left heart failure in the PH-LHD group (Interventrieular septum dimension 1.716 mm, left ventricular internal end diastolic dimension 4.888 mm, left ventricular posterior wall thickness in diastole 1.749 mm, ejection fraction 76.917%). But there was no difference in lung tissue between the sham group and PH-LHD group as showed by HE staining. Our results showed that the expression of IL-6 and TNF-α was highly expressed in PH-LHD rats’ serum and pulmonary vein, which were further increased after 2.0 g tension was given and were decreased after SAC/MAPKs inhibitors treatment. Meanwhile, on day 25, immunohistochemistry analysis showed the expression of IL-6 and TNF-α was higher in the PH-LHD rats’ pulmonary vein than that in pulmonary artery and lung tissue, and these expressions in pulmonary vein of PH-LHD group were also higher than that in sham group. However, on day 35, IL-6 and TNF-α were all increased in the pulmonary veins, arteries and lung tissues. Besides, our results uncovered that SAC/MAPKs pathway were upregulating in PH-LHD rats’ pulmonary vein.

**Conclusion:**

In conclusion, pulmonary vein mechanical stretching exacerbated PH-LHD possibly through the SAC/MAPKs pathway and upregulating expression of IL-6 and TNF-α.

## Background

Pulmonary hypertension due to left heart disease (PH-LHD) is caused by left heart dysfunction. The pressure of the left atrium first increases, followed by increased pulmonary vein pressure and pulmonary vascular smooth muscle cell proliferation, ultimately leading to pulmonary hypertension (PH). Currently, there is a lack of effective clinical treatments, and drugs or surgery can only improve the symptoms. However, PH-LHD has high morbidity and mortality, and is the most common type of PH observed in the clini c[1]. Therefore, it is of great importance to study the mechanism of pulmonary vascular remodeling.

PH-LHD is a kind of hemodynamic abnormality. Mechanical stretching caused by blood flow is an important factor maintaining the structure and function of the pulmonary vasculature in animals. Excessive blood pressure can promote thickening of the middle-level smooth muscle of pulmonary vascular and ultimately lead to pulmonary vascular remodeling. Current studies on PH-LHD have mostly focused on pulmonary artery, but Ahmed UF found that the severity of pulmonary hypertension was related to pulmonary vein remodeling, and pulmonary vein remodeling occurred earlier than pulmonary artery remodelin g[2]. Ping YX also showed that pulmonary veins first underwent arterialized changes during the progression of P H[3].

Interleukin-6 (IL-6) can promote the proliferation and differentiation of a variety of cells, and has strong proinflammatory effect s[4] Tumor necrosis factor-α (TNF-α) may affect pulmonary artery smooth muscle cell apoptosis by decreasing pyruvate dehydrogenase activit y[5]. The increasing of IL-6 and TNF-α is a reactive tissue repair mechanism. During pulmonary hypertension, pulmonary vascular and surrounding tissues secrete a large amount of IL-6, promoted the proliferation of pulmonary vascular smooth muscle cells and endothelial cell s[6]. In addition, TNF-α inhibitors have been shown to reduce the incidence of monocline-induced pulmonary hypertensio n[7]. However, the mechanism by which IL-6 and TNF-α involved in pulmonary hypertension formation is unclear.

We hypothesized that mechanical stretching of pulmonary veins mediates pulmonary vascular remodeling by activating the stretch-activated channel (SAC)/ mitogen-activated protein kinases (MAPKs) pathway, and upregulating IL-6 and TNF-α expression in the early stage of PH-LHD disease.

## Materials and methods

### Materials and animals

SB203580 (p38 MAPK inhibitor, 22,898), SP600125 (JNK1 inhibitor, 13,701), and U0126 (ERK1/2 inhibitor, 19,826) were bought from MedChem Express (NJ, USA). P38 MAPK Rabbit monoclonal antibody(1:1000, 8690), Phospho-p38 MAPK (p-p38 MAPK) Rabbit monoclonal antibody(1:1000, 4511), ERK1/2 MAPK Rabbit monoclonal antibody(1:1000, 4695), Phospho-ERK1/2 (p-ERK1/2) MAPK Rabbit monoclonal antibody(1:2000, 4370), JNK1 Rabbit monoclonal antibody(1:1000, 9252), and Phospho-JNK1 (p-JNK1) Rabbit monoclonal antibody(1:1000, 4668) were bought from Cell Signaling Technology. Rabbit IL-6 Polyclonal Antibody was bought from Bioss (1:500, bs-0782R, Beijing, China), Rabbit TNF-α antibody was bought from Abcam (1:1000, ab205587, USA). Peroxidase-conjugated goat anti-rabbit IgG(H + L) was purchased from Bioss (1:5000, bs-40295G, Beijing, China). Sixty male Sprague-Dawley (SD) rats (3–4 weeks, 80-100 g) were bought from Shanghai SLAC Laboratory Animal Co. Ltd. (License No. SCXK (HU)2017–0005). All rats were raised in a barrier environment of light/darkness (12/12 h alternations) at 22 °C ~ 24 °C, and were provided with adequate food and water. All experimental protocols are consistent with Institute of Laboratory Animal Resources, National Academy Press, Washington, DC1996.

### Experimental group

Sixty male SD rats were randomly divided into the sham group (including S1-S3 groups, *n* = 6) and the PH-LHD group (including M1-M7 group, n = 6) according to different inhibitors and mechanical tension. S1), S3): Mechanical tension: 0 g, 60 min. S2): Mechanical tension: 2.0 g, 60 min. M1), M7): Mechanical tension: 0 g, 60 min.: M2): Mechanical tension: 2.0 g, 60 min. M3): Mechanical tension: 2.0 g, 60 min, SB203580 (p38 MAPK inhibitor) pretreatment for 60 min. M4): Mechanical tension: 2.0 g, 60 min, SP600125 (JNK1 inhibitor) pretreatment for 60 min. M5): Mechanical tension: 2.0 g, 60 min, U0126 (ERK1/2 inhibitor) pretreatment for 60 min. M6): Mechanical tension: 2.0 g, 60 min, Streptomycin (SAC inhibitor) pretreatment for 60 min. SB203580, SP600125, U0126 and Streptomycin were dissolved in dimethyl sulfoxide (DMSO, HY-10999 MedChem Express, USA), and diluted with K-H solution to 200 μmol/L, 200 μmol/L, 200 μmol/L, and 1000 μmol/L at 37 °C, respectivel y[8].

### PH-LHD model establishment and echocardiography

PH-LHD model was established by banding procedure described by Siegfried B[9]. Three to four weeks old rat’s anesthesia was performed by intraperitoneal injection of 1.5% pentobarbital sodium (50 ml/kg) and placed on a ventilator. The thorax was opened at the second intercostal space on the left side of the sternum to expose the ascending aorta. A metal clip with an inner diameter of 0.8 mm was placed on the ascending aorta to cause the constriction of the ascending aorta. The sham group received a similar operation to expose the ascending aorta without clamped.

On day 25, the ultrasonic diagnostic instrument (GE VIVID-7 DIMENSION) equipped with a 10S probe (11.5 MHz, 200 cm/s, depth 2.5 cm) was used. After anesthesia, the probe was placed on the left sternum. The long axis section of the left ventricle point of the probe was placed at about 11 o ‘clock. Interventrieular septum dimension (IVSd), left ventricular posterior wall thickness in diastole (LVPWd), and left ventricular internal end diastolic dimension (LVIDd), ejection fraction (EF), heart rate (HR) and other data were measured. All data were averaged over 3 consecutive cardiac cycles.

### Vascular mechanical stretching

**Fig. 1:**
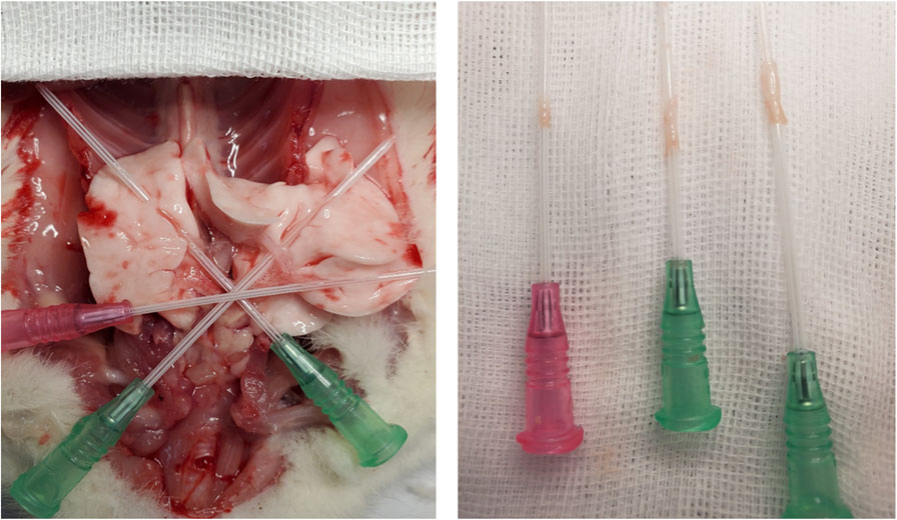
Exposed the pulmonary vein by puncture trocar

On day 25, after rats were sacrificed, the heart was removed, then exposed the pulmonary vein by puncture trocar as Fig. 1 showed. The pulmonary veins, pulmonary artery and lung tissues of the rats in S1-S2, M1-M6 groups were rapidly separated and placed in a K-H solution at 4 °C. Peripheral tissues of pulmonary veins were removed under microscope, and pulmonary veins were cut into 4 mm vascular rings. Then, pulmonary vein ring was hanged on the stainless-steel hook for mechanical stretching by a biofunction experiment system (620 M, Danish Myo Technology A/S, Denmark), and data were collected by the biological system (BL-420S, Chengdu Techman Software, China). During the experiment, the K-H solution was changed every 15 min, and a continuous mixture of 95% O_2_ and 5% CO_2_ was provided. Then a mechanical tension of 2.0 g was applied for 60 min, while in the S1 and M1 groups no mechanical tension was applied and inhibitors were added in the M3-M6 group. Finally, the above treated pulmonary vein rings were collected and stored in a ^− 80^°C freezer. Rats in S3 and M7 were kept alive until 35 days. On day 35, The pulmonary veins, pulmonary artery and lung tissues in S3 and M7 groups were collected as above method without mechanical stretching.

### ELISA

On day 25, serum was collected and stored at − 80 °C. The serum adds biotinylated antibodies, horseradish peroxidase-labeled enzyme, then TMB coloration to detect the expression of IL-6 and TNF-α in serum. All experiments were repeated three times.

### Hematoxylin eosin (HE) staining

On day 25, the lung tissue was fixed with 4% paraformaldehyde solution, dehydrated with ethanol, transparent with xylene and paraffin-embedded. Slices with a thickness of 4 m were cut, then stained with hematoxylin 5 min, differentiated with hydrochloric acid 30s, and stained with eosin 3 min. Pulmonary artery medium thickening, vascular myogenesis and an increase number of new vessels of lung tissues from model group and sham group were observed under microscope (CKX41, Olympus, Japan).

### Immunohistochemical analysis (IHC)

On day 25, lung tissues and pulmonary veins slices were placed in an oven at 60 °C for 2 h. The slices were successively placed in xylene for 15 min, and then put slices in 100% alcohol for 5 min, 85% alcohol for 5 min, and 75% alcohol for 5 min. After 15 min of citric acid antigen repair, the slices were incubated in 3% hydrogen peroxide for 10 min, and in goat serum for 30 min. Subsequently, the slices were incubated in IL-6 or TNF-α antibody for 8 h at 4 °C, then the slices were incubated in the secondary antibody for 50 min. Finally, the slices were stained, dehydrated, transparentized. The expression of IL-6 and TNF-α in pulmonary veins, pulmonary artery and lung tissue were observed under a microscope.

### Western blotting

RIPA Lysis Buffer (10 mL/mg) and protease inhibitor was added into the lung tissue and pulmonary vein, and then grinded lung tissue and pulmonary vein by tissue grinder (70 Hz, 1 min, Servivebio, China). The tissue homogenate was placed in a centrifuge for 15 min (4 °C, 15000 Rpm). And the supernatant was taken to detect protein concentration by BCA method. Add the heated denatured protein to SDS-PAGE, after protein electrophoresis (80 V) for about 1 h and electrotransfer (300 mA) for 45 min, PVDF membrane was sealed in 7% skim milk at room temperature for 2 h. After that, the PVDF membrane was incubated in primary antibody at 4 °C for 8 h. Then, the PVDF membrane was incubated in secondary antibody at room temperature for 1 h. Subsequently, ECL luminescence drops were placed on the washed PVDF film, and put it into the chemiluminescence instrument (ChemiDoc™ Touch system, Bio-Rad, USA). The gray value was analyzed by “Quantity One”. GAPDH was used as the internal control.

### Statistical analysis

The experimental results were expressed as Mean ± Standard Error of Mean. T-test was used for comparison between two groups with homogeneity of variance, one-way ANOVA was used for comparison between multiple groups. *P* < 0.05 was considered as statistically significant. SPSS 25.0 was used for data analysis, and Graphpad Prism 8.0 was used for data plotting.

## Results

### PH-LHD model establishment

**Fig. 2:**
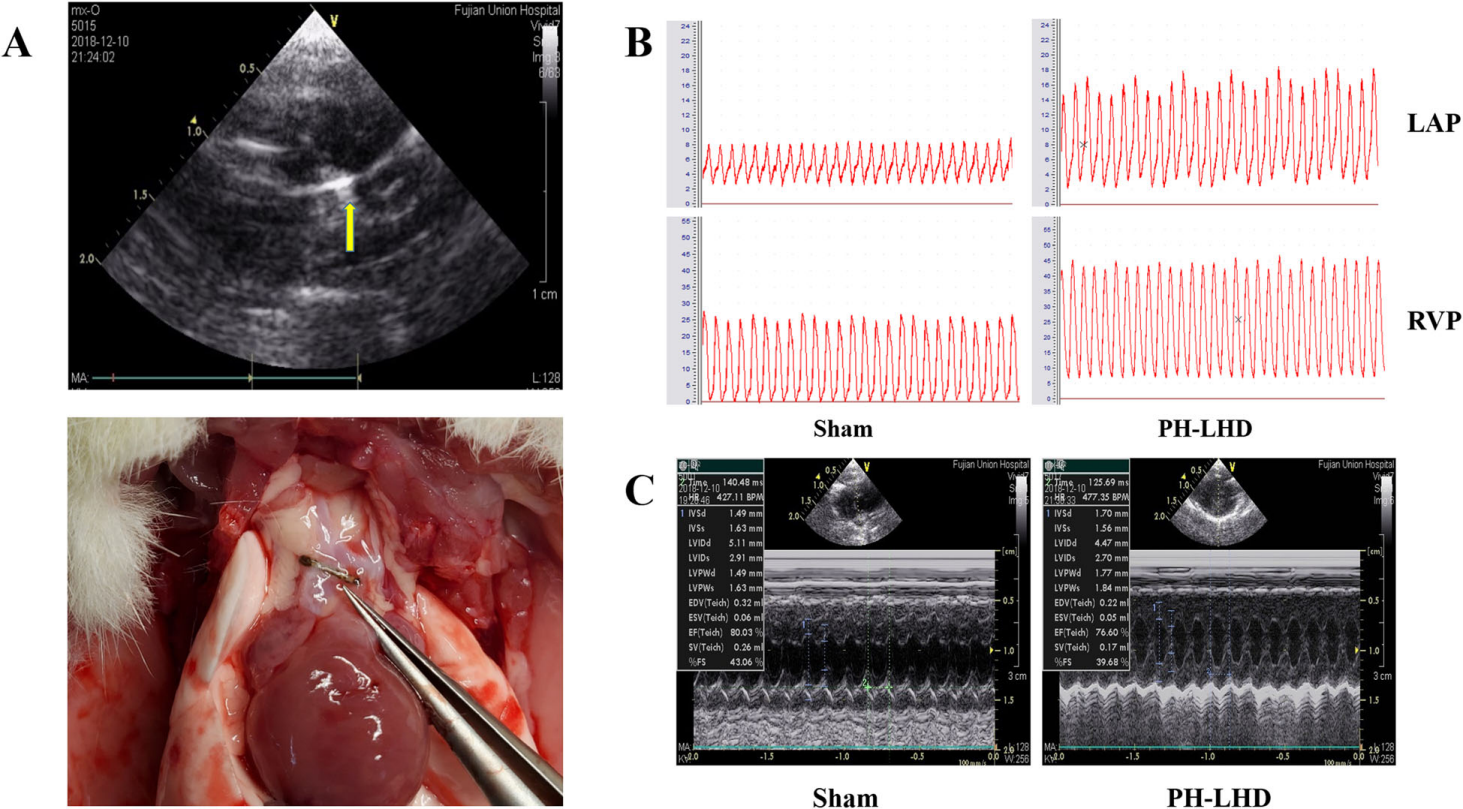
**a** Establishment of the PH-LHD model. The metal titanium clip (yellow arrow) was located at the ascending aorta of rats in the PH-LHD group by Doppler echocardiography. The PH-LHD model was established, and the position of the titanium clip was shown in the ascending aorta above the coronary artery. **b** RVP and LAP were determined in PH-LHD model rats. Representative images of RVP and LAP in sham and PH-LHD model rats are shown (*P* < 0.05). **c** Doppler echocardiography of left cardiac function in PH-LHD model rats. Representative images of left ventricular dysfunction in rats were assessed by Doppler echocardiography. **a** Sham model **b** PH-LHD model

On day 25, echocardiography showed no significant movement of the metal clamp (Fig. 2a). Right ventricular pressure (RVP) monitoring can effectively reflect pulmonary pressure, and left atrial pressure (LAP) monitoring can reflect the disease progression of PH-LHD. In this study, RVP and LAP in the PH-LHD group were higher than those in the sham group (Fig. 2b, Table 2) (*P* < 0.05). The ratios of heart weight (HW) to body weight (BW) and left ventricular (LV) + ventricular septum (VS)/right ventricular (RV) in PH-LHD group was greater than those in the sham group (Table 1) (*P* < 0.05). IVSD and LVPWd in the PH-LHD group were greater than that in the sham group, LVIDd and EF in PH-LHD group were less than those in the sham group (*P* < 0.05). There were no significant differences in heart rate (HR) between the PH-LHD group and the sham group (Fig. 2c, Table 2).

**Table 1:** Right ventricular pressure, left atrial pressure, HW/BW, (LV + V)/RV contrast

Group, *P* value	Sham group (*n* = 12)	PH-LHD group (*n* = 36)	*t* value	*P* value
RVP (cmH_2_0)	28.417 ± 3.034	35.339 ± 4.866	−6.325	*≤*0.001*
LAP (cmH_2_0)	8.227 ± 0.321	13.657 ± 2.402	−16.119	*≤*0.001*
HW/BW (%)	0.197 ± 0.016	0.212 ± 0.022	−2.340	0.022*
(LV + VS)/RV	2.827 ± 0.119	3.082 ± 0.061	−3.303	*≤*0.001*

*P ≤ 0.05

**Table 2:** Comparison of left ventricular function between the sham group and PH-LHD group

Group, *P* value	Sham group (n = 12)	PH-LHD group (n = 36)	*t* value	*P* value
HR (bpm)	404.421 ± 10.574	402.833 ± 21.494	0.307	0.760
IVSd (mm)	1.617 ± 0.137	1.716 ± 0.162	−2.349	0.022*
LVIDd (mm)	5.027 ± 0.200	4.888 ± 0.189	2.674	0.009*
LVPWd (mm)	1.643 ± 0.157	1.749 ± 0.148	−2.593	0.012*
EF% (mm)	81.198 ± 0.061	76.917 ± 1.833	16.163	<0.001*

*P ≤ 0.05

### HE staining

**Fig. 3:**
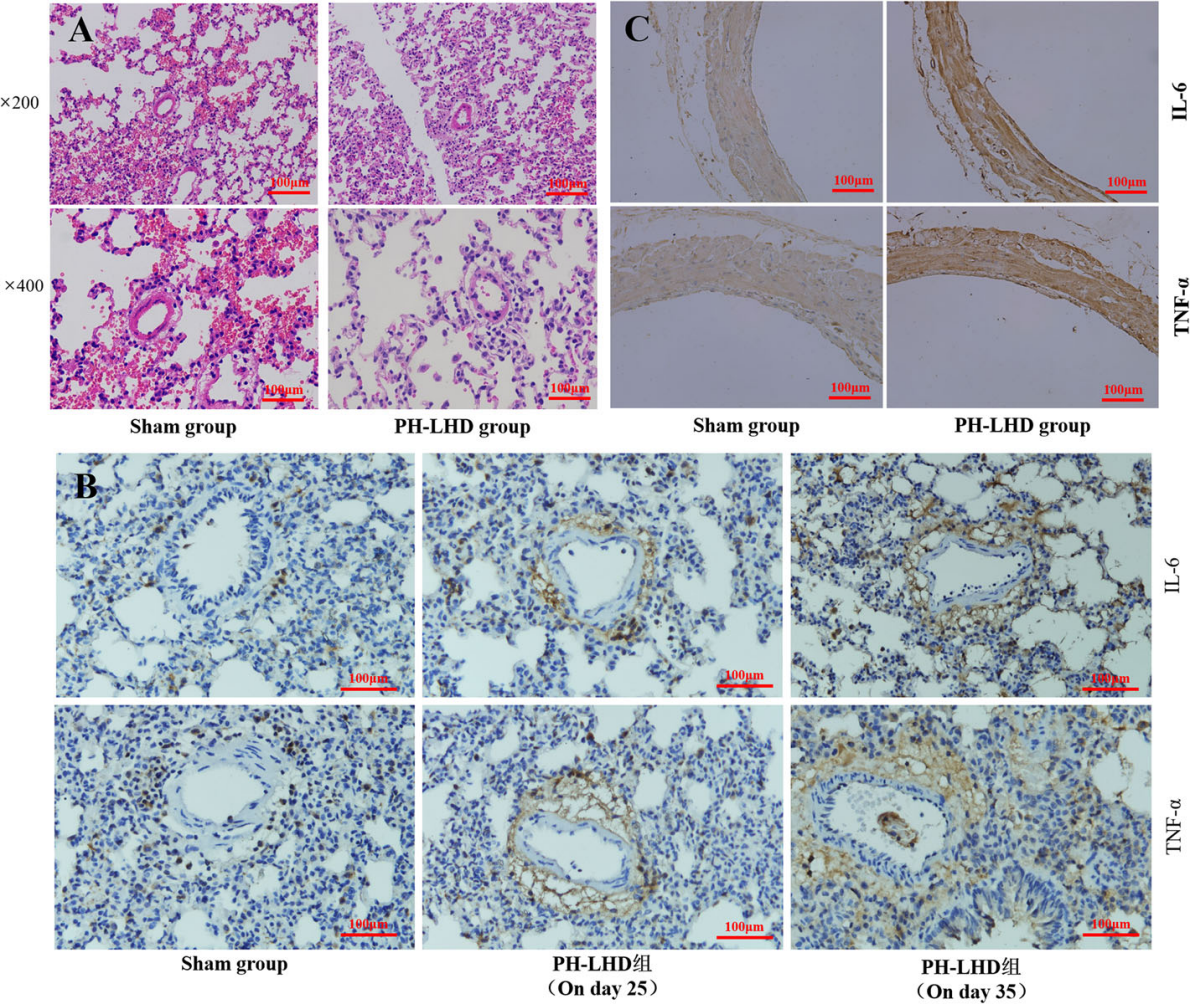
Pathological changes in lung tissues in PH-LHD model rats. **A.** Pathological structures were confirmed by HE staining in sham and PH-LHD model rats. **B.** IHC experiment was performed to examine the levels of IL-6 and TNF-α in the lung tissues of sham and PH-LHD model rats on day 25 and 35. **C.** IHC experiments were performed to examine the levels of IL-6 and TNF-α in the pulmonary veins of PH-LHD model rats

On day 25, HE staining was performed on the lung tissues of rats in the PH-LHD group and the sham group (Fig. 3a). There was no pulmonary hypertension induced pulmonary vascular remodeling, such as small pulmonary artery medium thickening, vascular myogenesis or an increase number of new vessels.

### Immunohistochemical staining

On day 25, IHC analysis of the lung tissue of rats in the S1, S2 and M1-M6 group showed that the expression of IL-6 and TNF-α around pulmonary veins was higher than that in the pulmonary artery and lung tissue in the PH-LHD group. However, there was no significant difference in the expression of IL-6 and TNF-α in the pulmonary vein, pulmonary artery and lung tissues in the sham group (Fig. 3b). On day 35, IHC analysis of the lung tissues of rats in the S3 and M7 groups showed that the expression of IL-6 and TNF-α around the pulmonary vein, pulmonary artery and lung tissue was all increased in the PH-LHD group (Fig. 3b).

On day 25, IHC analysis of pulmonary veins of rats in the S1, S2 and M1-M6 group showed that the expression of IL-6 and TNF-α in the PH-LHD group was higher than those in the sham group (Fig. 2c).

### Levels of IL-6 and TNF-α

**Fig. 4:**
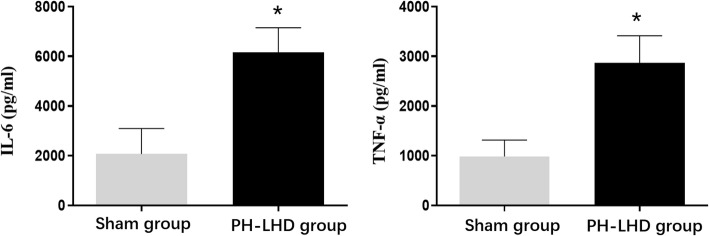
The concentrations of IL-6 and TNF-α in the serums of sham and PH-LHD model rats were analyzed by ELISA, *P < 0.05 vs. the sham group

**Fig. 5:**
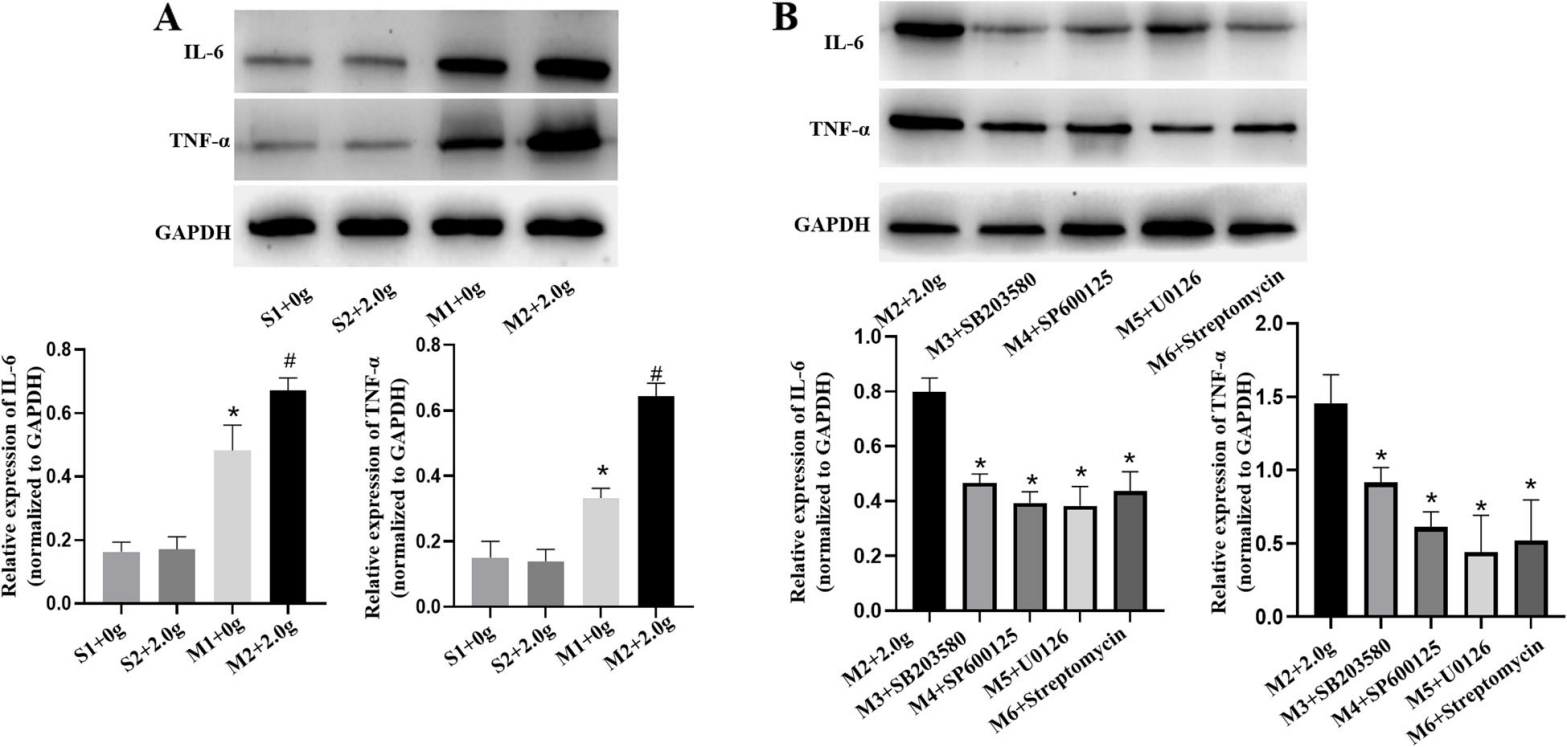
**a.** The protein expression levels of IL-6 and TNF-α were assessed by western blotting analysis in sham (0 g, 2.0 g) and PH-LHD model (0 g, 2.0 g) rats’ pulmonary vein, *P < 0.05 vs. S1, # *P* < 0.05 vs. M1. **b.** The protein expression levels of IL-6 and TNF-α in PH-LHD model rats’ pulmonary vein treated with SAC/MAPKs inhibitors were assessed by western blotting analysis, *P < 0.05 vs. M2

The serum and protein levels of IL-6 and TNF-α were determined by ELISA and western blotting. The expression of IL-6 and TNF-α in the PH-LHD group serum was higher compared with that in sham group serum (*P* < 0.05) (Fig. 4). Similar results were found in western blotting. The protein expression of IL-6 and TNF-α in M2 group which were enhanced by mechanical stretching were much higher compared with those in S1, S2 and M1 group (*P* < 0.05) (Fig. 5a). However, these expressions in the M2 group were downregulated by the addition of SB203580, SP600125, U0126 and streptomycin, as shown in Fig. 5b (*P* < 0.05).

**Fig. 6:**
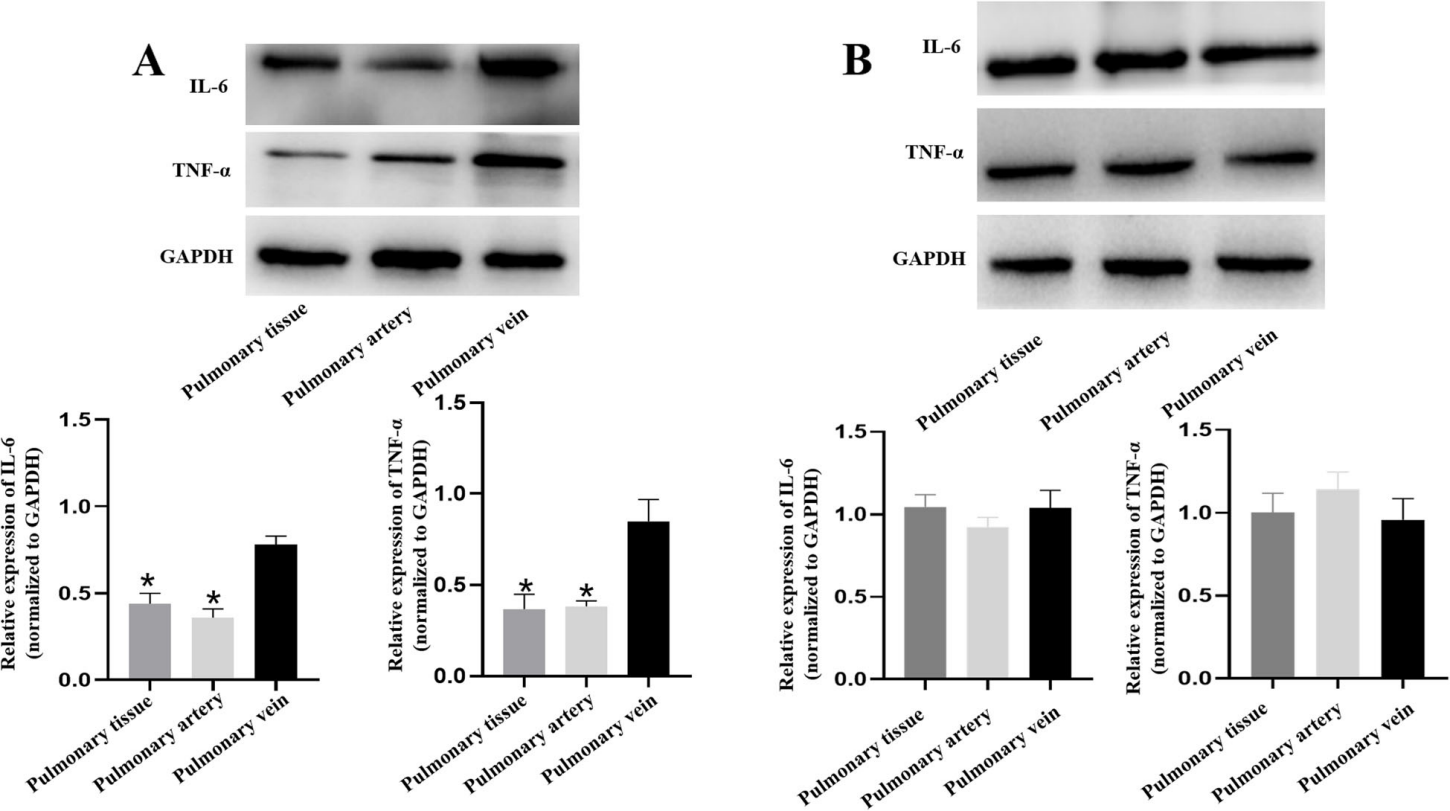
**a.** The protein expression levels of IL-6 and TNF-α in pulmonary vein, artery and lung tissue of the PH-LHD model on day 25 were assessed by western blotting analysis, *P < 0.05 vs. the pulmonary vein. **b.** The protein expression levels of IL-6 and TNF-α in pulmonary vein, artery and lung tissue of the PH-LHD model on day 35 were assessed by western blotting analysis

On day 25, western blotting in PH-LHD rat’s pulmonary vein, pulmonary artery and lung tissue showed that the expression of IL-6 and TNF-α in the pulmonary vein was increased compared with that in the pulmonary artery and lung tissue (*P* < 0.05) (Fig. 6a). However, on day 35, the expression of IL-6 and TNF-α was all increased in pulmonary vein, pulmonary artery and lung tissue (*P* < 0.05) (Fig. 6b).

### Levels of p-p38, p38, p-JNK1, JNK1, p-ERK1/2 and ERK1/2

**Fig. 7:**
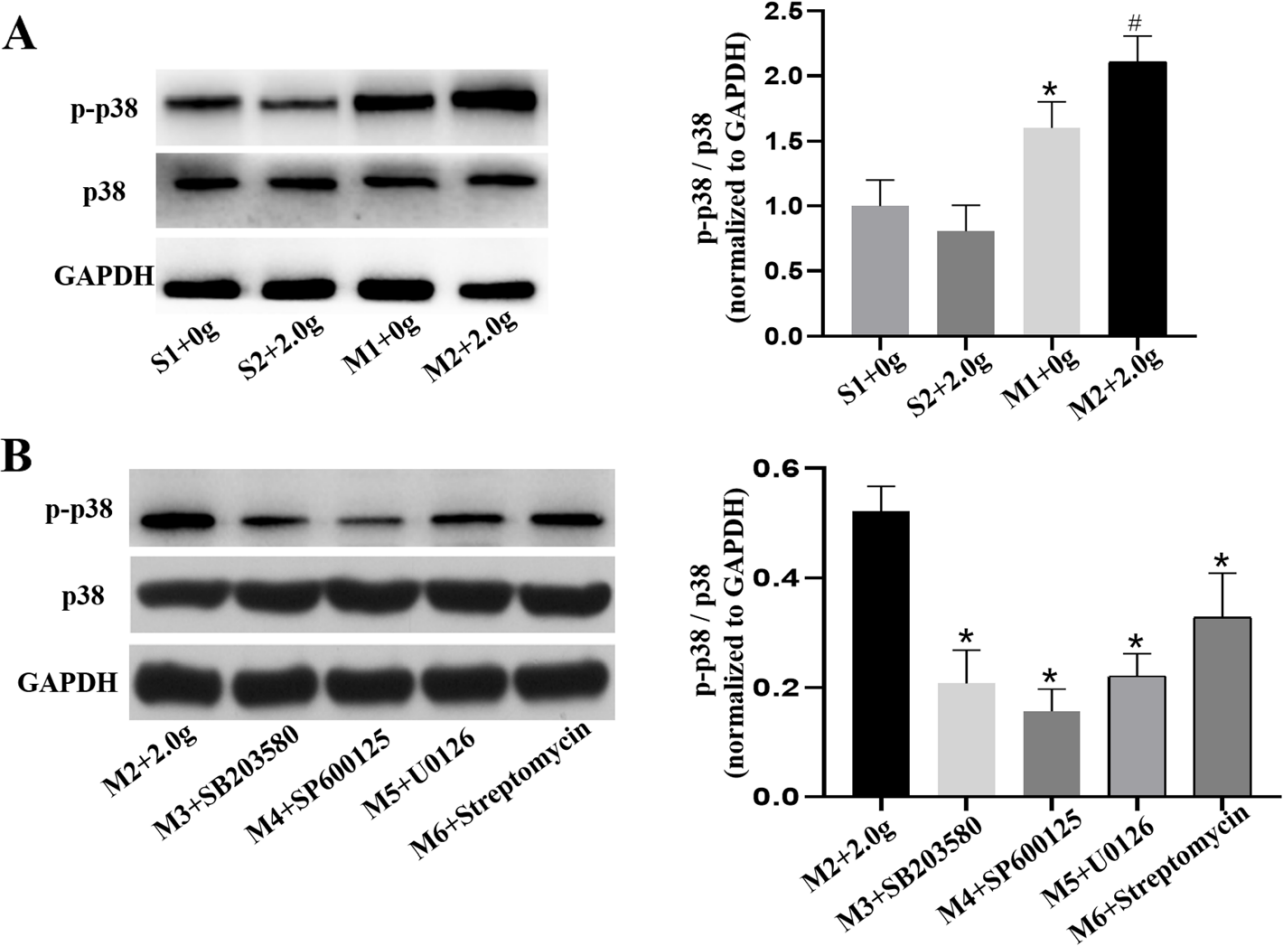
**a.** The protein expression levels of p-p38/p38 in the pulmonary veins of sham and PH-LHD model were assessed by western blotting analysis. *P < 0.05 vs. S1, # *P* < 0.05 vs. M1. **b.** PH-LHD model rats were treated with SB203580, SP600125, U0126 and streptomycin. *P < 0.05 vs. the M2

**Fig. 8:**
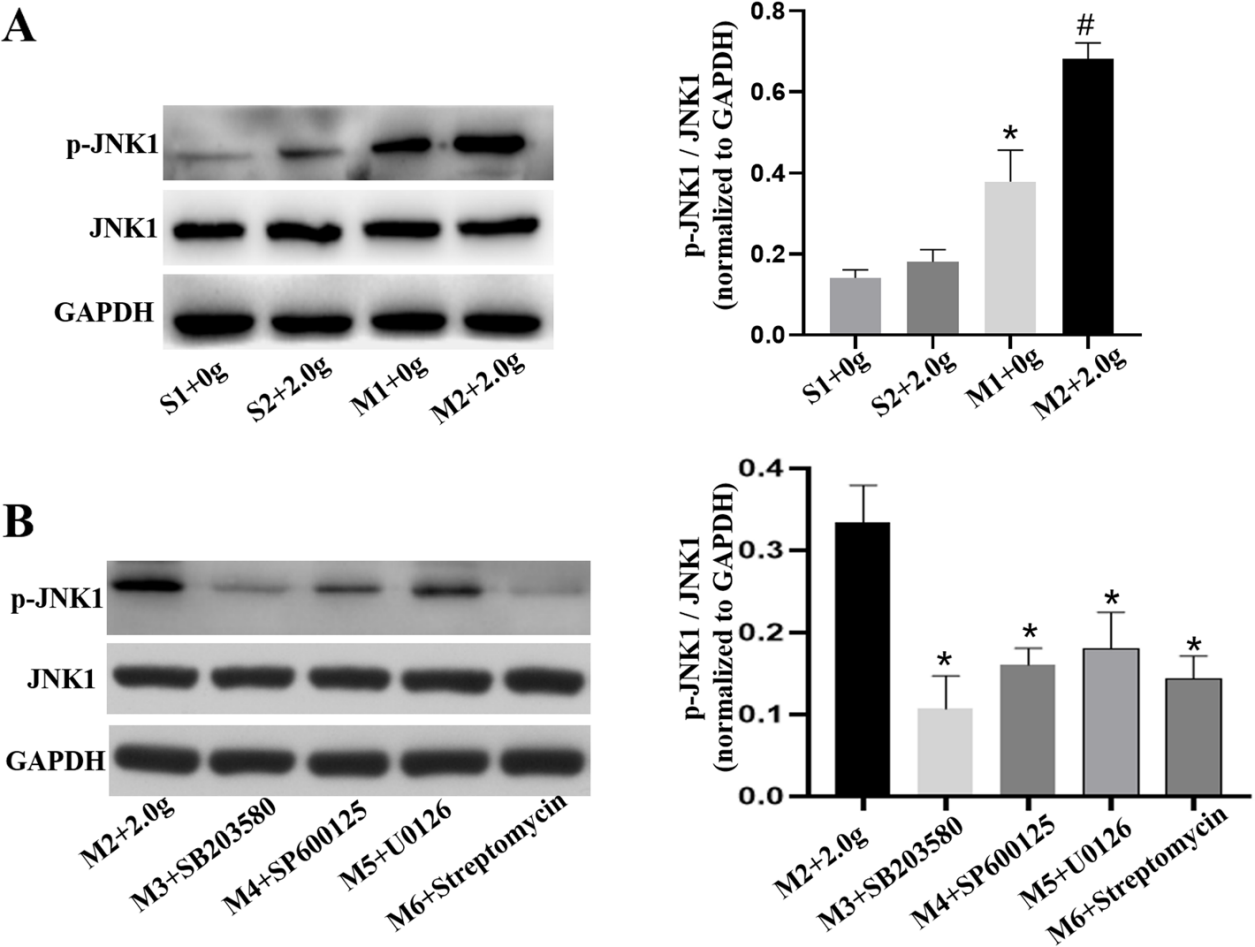
**a** The protein expression levels of JNK1 in the pulmonary vein of sham and PH-LHD model rats were assessed by western blotting analysis. *P < 0.05 vs. the S1, #*P* < 0.05 vs the M1. **b** PH-LHD model rats were treated with SB203580, SP600125, U0126 and streptomycin. *P < 0.05 vs. M2

**Fig. 9:**
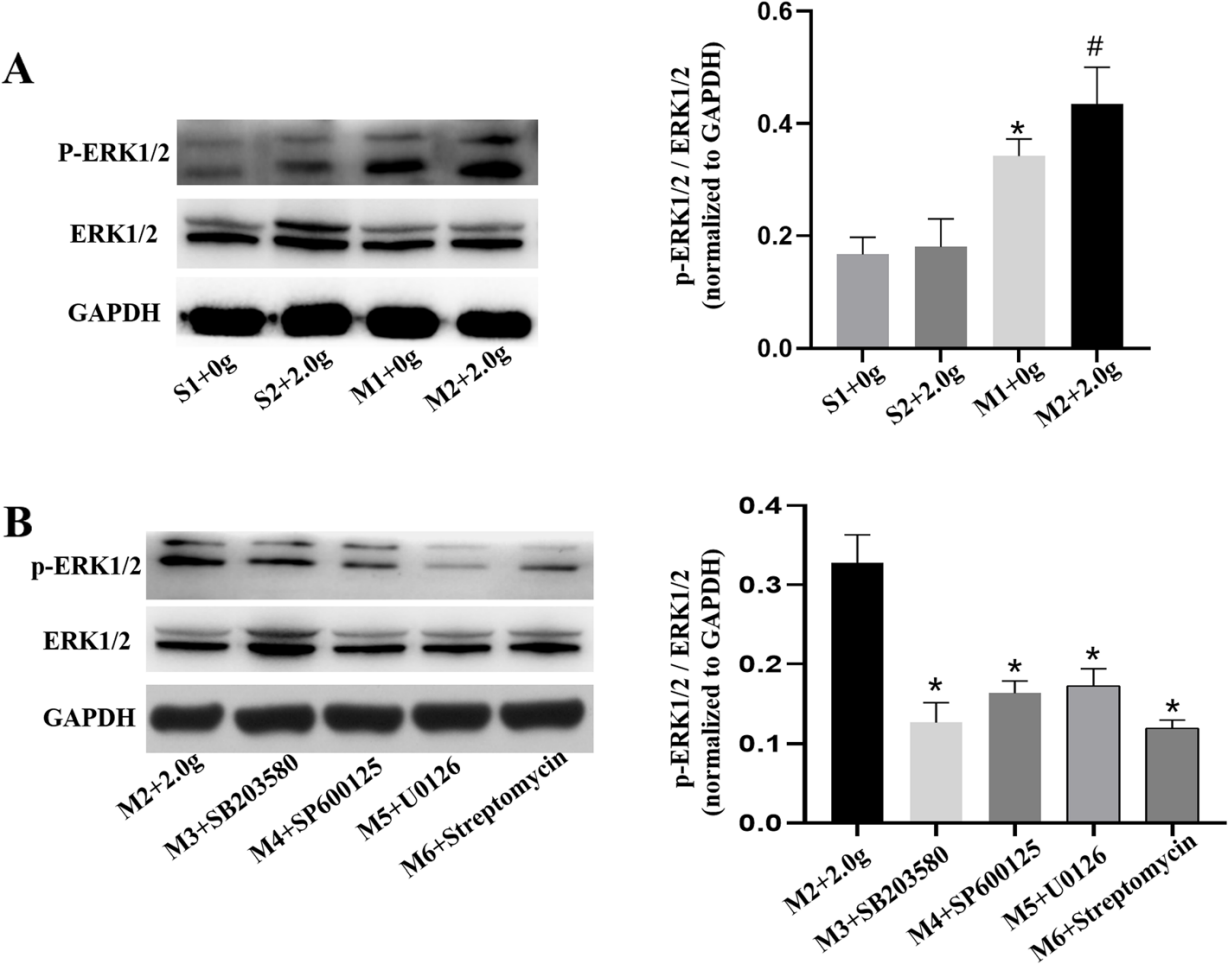
**a**. The protein expression levels of ERK1/2 in the pulmonary vein of sham and PH-LHD model rats were assessed by western blotting analysis. *P < 0.05 vs. S1, #*P* < 0.05 vs the M1. **b** PH-LHD model rats were treated with SB203580, SP600125, U0126 and streptomycin. *P < 0.05 vs. M2

The protein levels of p-p38, p38, p-JNK1, JNK1, p-ERK1/2 and ERK1/2 in pulmonary vein were determined by western blotting. Regardless of mechanical stretching, inhibitors addition or banding procedures, there were no obvious differences in the expression of p38, JNK1 and ERK1/2. The ratios of p-p38 to p38, p-JNK1 to JNK1 and p-ERK1/2 to ERK1/2 in M2 group were increased compared with those in S1, S2 and M1 group (*P* < 0.05) (Figs. 7, 8, 9). However, these ratios were reduced upon MAPKs or SAC inhibitors exposure, as shown in Figs. 7, 8, 9 (*P* < 0.5). Therefore, we revealed that mechanical stretching may be related to the increased expression of IL-6 and TNF-α in PH-LHD rats, which might through SAC/MAPKs pathway.

## Discussion

PH-LHD is characterized by enhanced pulmonary vascular resistance and pulmonary artery pressure. Currently, only symptomatic treatment is available for PH-LHD, and there is a lack of an effective radical cure. According to hemodynamics, the pulmonary vein is the first pulmonary vasculature to exhibit pressure changes in PH-LHD, and studies have shown that pulmonary vein remodeling occurs earlier and more seriously than pulmonary artery remodelin g[3]. At present, most studies on the mechanism of PH-LHD focus on the pulmonary artery, while this study focuses on the pulmonary vein to explore the early pathogenesis and therapeutic targets of PH-LHD.

Previous studies confirmed that 15 days after banding procedure, right ventricular pressure began to increase, and vascular remodeling with middle vascular thickening and lumen stenosis occurred on day 5 0[10]. In this study, on day 25, left heart weight was increased, and appearance of left ventricular afterload increased, leading to myocardial hypertrophy and reduced left ventricular inner diameter were occurred in echocardiography. Despite LAP and RVP increasing changed cardiac structure in the PH-LHD group, the HE staining results showed no significant difference in lung tissue morphology between the two groups. Therefore, the 25 days after the banding procedure was still in the early stage of PH-LHD progression, and was selected as the time point for observation of the pulmonary vein.

Pulmonary vascular remodeling is an important pathological manifestation of pulmonary hypertension. Studies have shown that the accumulation of inflammatory cells can lead to vascular remodeling,[11] and as potent proinflammatory factors, IL-6 and TNF-α have been shown to be elevated in various types of P H[12, 13]. IL-6 is a cytokine that alters the inflammatory response by producing acute phase proteins by affecting T-cell differentiation or B-cell transformation into plasma cells. Studies have shown that in the hypoxia-induced PH model, IL-6 mRNA and protein expression levels are increased,[14, 15] and pulmonary hypertension and right ventricular hypertrophy are improved in the IL-6 gene-knockout mous e[16]. TNF-α is an important inflammatory factor that regulates the growth and apoptosis of mammalian cells and is involved in various inflammatory pathological reactions. TNF-α may induce pulmonary vasoconstriction by promoting the expression of endothelin. In addition, TNF-α has been shown to promote the expression of interleukin and matrix metalloproteins in cardiopulmonary diseases, promoting vascular remodelin g[17–19]. In this study, 25 days after banding procedure, the expression of IL-6 and TNF-α in the serum and pulmonary vein in the PH-LHD group was higher than those in the sham group. Meanwhile, these expression in pulmonary veins were highest compared with pulmonary arteries and lung tissues. However, on day 35, these expressions were all increased in the pulmonary vein, pulmonary artery and lung tissue in the PH-LHD rats. It can be concluded that the expression of inflammatory factors in pulmonary veins occurred earlier than that in the pulmonary artery and lung tissue in the early stage of PH-LHD. As a result of the pressure load on the wall of the pulmonary vein, inflammatory cell accumulation occurs, and IL-6 and TNF-α are synthesized and released to maintain changes in vascular structure and function.

Stretch-activated channel (SAC) was initially discovered in skeletal muscle cells by Guharay^,^[20] and subsequent studies have shown that SAC is widely present in various cell s[21]. Qiu found that mechanical tension stimulates the SAC signaling pathway and regulates the expression of corresponding mRNAs and proteins in a rat aortic dissection mode l[22]. The pathophysiological similarity between PH-LHD and aortic dissection is that the vasculature is stimulated by mechanical tension which causes vascular remodeling. In this study, mechanical stretching of pulmonary veins in the sham group did not increase the expression of IL-6 and TNF-α. However, after administering appropriate mechanical tension to the pulmonary veins in PH-LHD group rats, the expression of IL-6 and TNF-α, which had already been increased compared with that of the sham group rats, was further increased due to mechanical stretching. Therefore, we hypothesized that in the PH-LHD rat model, mechanical tension on the vasculature activated some signaling pathways in cells and corresponding inflammatory cytokine expression.

Studies have shown that mechanical stretching is associated with some signaling pathways on the cell membrane and regulates pulmonary vascular remodeling. Shyu KG found that the gene encoding smooth muscle cell contractility protein was involved in pulmonary vascular remodeling due to the regulation of the stretch-induced RhoA pathway and related transcription factor s[23]. Jing Z found that mechanical stretching regulated the expression of CXCL1 and CX3CL1 genes which are associated with inflammation, by activating the STAT1 signaling pathway, thereby inhibiting vascular remodelin g[24].

In addition, studies have shown that the AKT, ROS and NF-κB signaling pathways are involved in mechanical stretching-induced vascular remodelin g[25–27]. The MAPKs pathway is one of the main signaling pathways in organisms and can regulate the expression of inflammatory responses and cytokines. MAPKs have been proven to be divided into three major signaling pathways. The ERK1/2 signaling pathway can regulate cell growth and differentiation, and the JNK and P38 pathways play important roles in inflammation and apoptosi s[28–31]. Liu X demonstrated that the MAPK-JNK pathway can respond to mechanical tension stimulation, and then phosphorylate and induce vascular remodelin g[32]. SB203580, SP600125 and U0126 are inhibitors of p38, JNK1 and ERK1/2 pathways respectively. SB203580 directly inhibits the p38 pathway in an ATP-competitive manne r[33]. SP600125 reversibly and competitively binds to the JNK anthrazolinone domain,[34] inhibits JNK pathway activity and regulates cell apoptosis. U0126 antagonizes the transcriptional activity of activated protein 1 (ap-1) and inhibits the activation of MAPK p42 and p44 by noncompetitive means, thus, the activation of ERK1/2 is significantly blocked by U012 6[35]. Streptomycin inhibits the SAC pathway. To confirm the correlation between IL-6 and TNF-α expression and the SAC/MAPKs pathway, SB203580, SP600125, U0126 and streptomycin were used to treat the rats. The results showed that p-p38, p-JNK1 and p-ERK1/2 protein was highly expressed in the pulmonary veins in the PH-LHD group, and these expressions were further increased by additional mechanical tension. However, similar trends were not found in the sham group. When SAC/MAPKs signaling pathway inhibitors were further used, the expression of p-p38, p-JNK1 and p-ERK1/2 was inhibited. Moreover, decreased expression of IL-6 and TNF-α in pulmonary veins also occurred after SB203580, SP600125, U0126 and streptomycin administration.

Thus, activation of the p38, JNK1 and ERK1/2 pathways in the pulmonary vein in PH-LHD model rats was increased, and the expression of these signaling pathways could be further upregulated by mechanical stretching of the pulmonary vein in vitro. SAC inhibitors also antagonized the increased expression of the MAPKs signaling pathway, thus we hypothesized that MAPKs pathway might induce channel protein activation through the up-regulation of SAC pathway. Through inhibition of the p38, JNK1, ERK1/2 and SAC signaling pathways, the expression of IL-6 and TNF-α caused by mechanical stretching of the pulmonary vein could be decreased. When the pulmonary vein is stimulated by appropriate mechanical tension, the SAC pathway can be stimulated, activating the MAPKs pathway and upregulating the expression of inflammatory factors. In addition, since SP600125, SB203580 and U0126 inhibit the activation of p38, JNK1 and ERK1/2 pathways, respectively, by regulating ATP activity,[36, 37] all branches of MAPKs pathway are directly inhibited by these inhibitors, and the expression of factors of the other two pathways can be downregulated to a small extent.

## Conclusions

In the early stage of PH-LHD, the inflammatory response is involved in pulmonary vascular remodeling, the duration of pulmonary vein inflammation and the expression of related factors occurs earlier than in the pulmonary artery and lung tissue. Pulmonary vein mechanical stretching may mediate increased expression of IL-6 and TNF-α through the SAC/MAPKs pathway, which provides a new target for future clinical treatment.

### Limitation

On day 35, morphological changes and protein expression in rat pulmonary vein should be more verified. It will continue in future experiments.

## Data Availability

Data used for this study are available upon request.

## Abbreviations

RVP: Right ventricular pressure
LAP: Left atrial pressure
IVSD: Interventrieular septum dimension
LVPWd: Left ventricular posterior wall thickness in diastole
LVIDd: Left ventricular internal end diastolic dimension
EF: Ejection fraction
HR: Heart rate
MAPK: Mitogen-activated protein kinase
PH-LHD: Pulmonary hypertension due to left heart disease
SAC: Stretch-activated channel
