# Correction to: Mechanical stretching of the pulmonary vein mediates pulmonary hypertension due to left heart disease by regulating SAC/ MAPK pathway and the expression of IL-6 and TNF-α

**DOI:** 10.1186/s13019-021-01554-3

**Published:** 2021-06-09

**Authors:** Wenhui Huang, Hongjin Liu, Yichao Pan, Hongwei Yang, Jing Lin, Hui Zhang

**Affiliations:** 1 Department of Cardiovascular Surgery, Union Hospital, Fujian Medical University, Fuzhou, 350001 Fujian Province People’s Republic of China; 2 Anesthesiology Research Institute, the First Affiliated Hospital, Fujian Medical University, Fuzhou, 350004 Fujian Province People’s Republic of China; 3 Department of Intensive Care Unit, Union Hospital, Fujian Medical University, Fuzhou, 350004 Fujian Province People’s Republic of China

**Correction to: J Cardiothorac Surg 16, 127 (2021)**

**https://doi.org/10.1186/s13019-021-01471-5**

The original article [1] contained an incorrect designation of co-first authorship which has since been removed.
